# Global measurement of coagulation in plasma from normal and haemophilia dogs using a novel modified thrombin generation test – Demonstrated *in vitro* and *ex vivo*

**DOI:** 10.1371/journal.pone.0175030

**Published:** 2017-04-06

**Authors:** Daniel Elenius Madsen, Timothy C. Nichols, Elizabeth P. Merricks, Emily K. Waters, Bo Wiinberg

**Affiliations:** 1 Translational Haemophilia Pharmacology, Global Research, Novo Nordisk A/S, Måløv, Denmark; 2 Department of Pathology and Laboratory Medicine, University of North Carolina, Chapel Hill, North Carolina, United States of America; 3 Haemophilia Biology, Global Research, Novo Nordisk A/S, Måløv, Denmark; University of Pennsylvania Perelman School of Medicine, UNITED STATES

## Abstract

**Introduction:**

Canine models of severe haemophilia resemble their human equivalents both regarding clinical bleeding phenotype and response to treatment. Therefore pre-clinical studies in haemophilia dogs have allowed researchers to make valuable translational predictions regarding the potency and efficacy of new anti-haemophilia drugs (AHDs) in humans. To refine *in vivo* experiments and reduce number of animals, such translational studies are ideally preceded by *in vitro* prediction of compound efficacy using a plasma based global coagulation method. One such widely used method is the thrombin generation test (TGT). Unfortunately, commercially available TGTs are incapable of distinguishing between normal and haemophilia canine plasma, and therefore *in vitro* prediction using TGT has so far not been possible in canine plasma material.

**Aim:**

Establish a modified TGT capable of: 1) distinguishing between normal and haemophilia canine plasma, 2) monitoring correlation between canine plasma levels of coagulation factor VIII (FVIII) and IX (FIX) and thrombin generation, 3) assessing for agreement between compound activity and thrombin generation in *ex vivo* samples.

**Methods:**

A modified TGT assay was established where coagulation was triggered using a commercially available activated partial thromboplastin time reagent.

**Results:**

With the modified TGT a significant difference was observed in thrombin generation between normal and haemophilia canine plasma. A dose dependent thrombin generation was observed when assessing haemophilia A and B plasma spiked with dilution series of FVIII and FIX, respectively. Correlation between FVIII activity and thrombin generation was observed when analyzing samples from haemophilia A dogs dosed with canine FVIII. Limit of detection was 0.1% (v/v) FVIII or FIX.

**Conclusion:**

A novel modified TGT suitable for monitoring and prediction of replacement therapy efficacy in plasma from haemophilia A and B dogs was established.

## Introduction

Haemophilia A and B (HA and HB, respectively) are severe bleeding disorders caused by mutations in the genes encoding coagulation factor VIII (FVIII) or coagulation factor IX (FIX), respectively. Patients present with low or undetectable plasma activities of FVIII or FIX, leading to a decreased capacity to form thrombin. Clinically this decreased capacity manifests as spontaneous bleeding episodes affecting the joints, muscles and soft tissue[1]. Disease severity, typically based upon residual plasma activity of FVIII or FIX, is classified as mild (<40%), moderate (<5%) or severe (<1%) [2]. The classification of HA patients based upon residual factor activity is well-documented, especially for classification of HA patients with a clinically severe bleeding phenotype[3]. The standard treatment regimen for haemophilia is prophylactic or on-demand replacement therapy with FVIII or FIX, but treatment is complicated by a risk of developing inhibitory antibodies towards the administered coagulation factors. For FVIII replacement therapy, approximately 30% of patients develop inhibitors against FVIII, and much effort has been and is still put into development of anti-haemophilic drugs (AHD) that bypass FVIII and FIX in the coagulation system[4].

Globally different treatment protocols are used for treating haemophilia[5], where some regimens aim at obtaining a certain factor trough level, and others on tailoring the treatment according to individual bleeding phenotype. It is however difficult, to predict the exact dose of FVIII or FIX needed to stop or prevent bleeding episodes in individual patients. This makes optimizing haemophilia treatment based on individual needs challenging[6], and improved methodologies are needed to address these issues. Evidence is accumulating to support the hypothesis that the thrombin generation test (TGT) may reflect the bleeding risk of patients with coagulation disorders[7]. By utilizing the TGT researchers have reported correlations between TGT parameters and the clinical bleeding phenotype as well as response to treatment in human HA patients [8, 9].

Haemophilia A and B dogs are excellent models of haemophilia, as clinical bleeding phenotype and response to treatment resembles their human equivalents[10, 11] and canine models of haemophilia have served as valuable tools for assessing the potency of AHD for decades[11]. As in humans, it is unpredictable how much FVIIa, FVIII or FIX is needed to stop or prevent bleeding episodes in individual dogs, and new tools for monitoring treatment in these dogs is therefore desirable for monitoring of treatment efficacy. In translational studies, where AHDs were tested in animal models, the TGT method proved useful for testing and qualification of new AHD candidates[12, 13]. However the standard TGT method is not sensitive to the levels of FVIII and FIX in the plasma of dogs, and is therefore not able to distinguish between normal and HA or HB dogs.

Thus, to rationally predict treatment efficacy in canine models of haemophilia both *in vitro* and *in vivo* we aimed to develop a canine optimized TGT. The assay should have the following attributes: 1) distinguish between normal and haemophilia dogs, 2) monitor correlation between plasma FVIII/FIX *in vitro* activity and thrombin generation and 3) assess for correlation between FVIII activity and thrombin generation in *ex vivo* samples.

## Materials and methods

### Ethical approval

All protocols carried out at University of North Carolina, Chapel Hill, NC, US were in accordance with institutional guidelines and approved by the Institutional Animal Care and Use Committee at the University of North Carolina at Chapel Hill (Approval number 14–111),

All studies were approved by Novo Nordisk A/S internal Ethical Review Committee.

### Husbandry, diet, housing and care of dogs at University of Chapel Hill, Francis Owen Blood Research Lab (FOBRL)

All of the dogs in this study were produced and maintained at the FOBRL at the University of North Carolina at Chapel Hill. The FOBRL is a specialized facility that houses strains of dogs with hemophilia A, hemophilia B, von Willebrand disease (VWD), combined hemophilia A/VWD, FVII deficiency, and normal blood donor dogs. Dogs are housed on indoor/outdoor runs, whereby the indoor portion provides conditioned space. In general, dogs are housed in pairs for socialization. This space for all animals meets or exceeds USDA and NIH requirements and AAALAC recommendations. In addition, the dogs are allowed into fenced play yards for socialization and enrichment. Two exam/procedure rooms are used for all examinations, clinical treatments and transfusions, blood collection and plasma banking, dental prophylaxis, and experimental procedures. Normal dogs are maintained as a “walking blood bank” for canine replacement products. Some of these animals are Universal donors to provide whole blood for direct transfusion in emergency cases. Clinical diagnostic equipment (e.g., microscopes, SCIL cell counter, otoscope, stethoscopes, refractometer, thermometers, hemoglobinometer) is on site. The animals are fed once or twice daily and their housing areas are cleaned at least once daily. Most of the dogs in the colony are fed solid food but some have special needs and require different source of nutrition. Nutrition is discussed regularly with the attending veterinarians and any changes are made accordingly. The dogs in this study have severe hemophilia. The bleeding phenotype is severe, spontaneous and stochastic and without prompt treatment can be crippling or fatal. All dogs are checked at least twice daily, 7 days a week for any health problem with a particular emphasis on the emergence of a bleeding event. This professional staff and registered veterinary technicians share call 24/7 for emergencies. Animal care at the FOBRL is designed and supervised by the veterinary staff of the Division of Laboratory Animal Medicine. Several of the members of this team have cared for these dogs for over 20 to 30 years.

### Monitoring of dogs used for *ex vivo* study

The dogs in this study had blood samples drawn by venipuncture. After sampling, they were monitored until hemostasis was confirmed, usually 10 to 15 minutes. Any issues related to pain or distress are discussed with the attending veterinarian. The dogs in this study only had blood samples drawn by venipuncture so any related pain or distress was minimal and very brief.

No dogs were euthanized for this study. All dogs were returned to the colony.

### Haemostatically normal and haemophilic dogs

Blood samples were collected from normal healthy Hound Mix dogs at FOBRL, UNC, Chapel Hill, NC.

Blood samples were collected from HA and HB dogs from the dog colony at FOBRL. The underlying genetic defect of the severe HA dog colony at Chapel Hill is due to an inversion in intron 22 of the FVIII gene. All dogs carrying this mutation present with undetectable levels of FVIII based upon activity measures (<0.3% FVIII activity)[14, 15]. The FVIII activity in all plasma samples drawn from HA dogs was measured using a chromogenic FVIII activity assay (COAMATIC FACTOR VIII, Chromogenix, Instrumentation Laboratory Company, Bedford, MA, US) according to manufacturer’s instructions, with normal canine plasma diluted in HA plasma as calibrator. The limit of detection of this method is 0.3% FVIII activity. Unless stated otherwise, the FVIII activity in the HA plasma samples were below the detection level of the assay.

The HB dog colony disease is caused by a point mutation in the FIX encoding gene, which affects the active site of the FIX protein, giving rise to undetectable plasma levels of FIX [15, 16].

### Blood drawing and plasma preparation

Blood samples were collected from at least 12h fasted dogs. Blood samples were drawn from the cephalic vein in 3.2% sodium citrate (1 part 3.2% sodium citrate and 9 parts blood). Plasma was obtained by double centrifugation of whole blood at 2200·G for 15 min followed by centrifugation of plasma at 2200·G for 7 min at 4°C. Aliquots were frozen at -80°C 5 minutes after aliquotation.

### Establishment of plasma pools

Three separate normal canine plasma pools were established from healthy hound mix dogs at FOBRL. The pools are denoted normal pool I, II and III.

Two HA and one HB canine plasma pools were established from five individual HA and HB dogs (referred to as HA pool I and II and HB pool).

A HA and HB canine plasma pool with 0.1% (v/v) normal plasma was prepared by addition of 0.1% (v/v) normal plasma to the HA or HB plasma pool (referred to as 0.1% norm. in HA and HB).

### Recombinant canine B-domain deleted FVIII (rcBDDFVIII)

Recombinant canine B-domain deleted FVIII (rcBDDFVIII) was produced as described by Sabatino et al.[17].

### Inhibitory capacity of an anti canine-FVIII polyclonal antibody (Anti cFVIII PAb)

A commercial available inhibitory polyclonal sheep anti cFVIII antibody SAC8C-IG (lot:Ig1386R4) (Affinity Biologicals, Ancaster, Ontario, Canada) was used throughout the experiments. The inhibitory capacity of this antibody was tested by incubating normal pool III with 0.1 mg/mL anti cFVIII PAb for 30 minutes at room temperature with mild agitation. The FVIII activity in this preparation was subsequently analyzed using a chromogenic FVIII activity assay (COATEST SP4 FVIII, Chromogenix, Instrumentation Laboratory Company, Bedford, MA, US) according to manufacturer’s instructions, but with normal pool III diluted in HA pool II as calibrator. All samples were run in duplicate.

### Thrombin generation test using the Calibrated Automated Thrombogram (CAT) method

Thrombin generation was measured using the CAT method, essentially as described by Hemker et al., but using half the volume of reagent and plasma [18]. Ten μL trigger solution PPP reagent (5 pM rhTF final in asssay) or PPP reagent low (1 pM rhTF final in assay)(Thrombinoscope BV), or 10 μL Thrombin calibrator (Thrombinoscope BV) was added to separate wells in Immulon 2HB 96-well round-bottom microtiter plates (Thermo Scientific, Waltham, MA, USA). Forty μL plasma, thawed at 37°C for 5 mins, was added to each well, and the plate was incubated for 10 mins at 37°C in a Fluoroskan Ascent fluorometer (Thermo Scientific). Forty μL Fluo Substrate (Z-gly-gly-arg-AMC, Thrombinoscope BV) was added to 1600 μL Fluo Buffer (Thrombinoscope BV) pre-warmed to 37°C, and 10 μL of this solution was automatically dispensed to each well using the fluorometer. The calcium source in this setup is the Fluo Buffer, which contains 100 mM CaCl_2_). A final assay concentration of 16.6 mM CaCl_2_ is therefore obtained. Thrombograms were obtained using the accompanying Thrombinoscope software (Thrombinoscope BV). From the thrombograms the following thrombin generation parameters were automatically calculated: lag time, endogenous thrombin potential (ETP), peak thrombin generation and time to peak (TTP).

### Assessment of the thrombin formation in canine plasma samples using the standard calibrated automated thrombogram (CAT) method

Normal pool III, normal pool III incubated with 0.1 mg/ml anti cFVIII PAb and HA pool II were analyzed using the standard CAT method, with PPP reagent and PPP reagent low (5 and 1 pM rhTF final dilution in assay, respectively). All samples were analyzed in quadruplicate.

### Thrombin generation test using the canine optimized TGT

Twenty μL BSA5-buffer (20 mM HEPES, 140 nM NaCl (Merck KGaA, Darmstadt, Germany), 5 g/L bovine serum albumin (Sigma Life Science, St. Louis, MO, US), pH 7.35) or 20 μL Thrombin calibrator was added to separate wells in Immulon 2HB 96-well microtiter plates. Seventy μL test plasma was added to each well. Forty-four μL Fluo Substrate were added to 1600 μL 37°C Fluo Buffer, and the ampoule was whirl-mixed for 5 sec. Immediately before initiating the experiments 230 μL Actin FS (Siemens Healthcare Diagnostics Product GmbH, Marburg, Germany) were added to the above mentioned solution, and the solution was whirl-mixed for 5 seconds. Subsequently this solution was loaded onto the apparatus’ pump, and 30 μL of this solution was automatically dispensed to each well. Thrombin generation parameters were obtained as mentioned above.

Using the canine optimized TGT a final assay concentration of 21.3 mM CaCl_2_ was obtained.

### Analysis of canine plasma material using the canine optimized TGT

#### Assessment of the capability of the canine optimized TGT to discriminate between normal and HA canine plasma

Plasma samples from five healthy dogs, five HA dogs, normal pool II and the HA pool were analysed using the canine optimized TGT.

To asses if residual amounts of FVIII, below the detection limit of the chromogenic FVIII activity assay, in individual HA plasma samples gave rise to variation in thrombin generation, plasma samples from five HA dogs were incubated with 0.1 mg/mL anti cFVIII PAb or PAb buffer for 30 min at RT with mild agitation, and were subsequently analyzed using the canine optimized TGT.

To confirm the capacity of the anti cFVIII PAb to inhibit thrombin formation in plasma normal pool II was analyzed in the presence and absence of 0.1 mg/mL anti FVIII PAb. The HA canine plasma pool was used as comparator.

#### Assessment of cFVIII and cFIX dose dependent thrombin generation

Normal pool I was diluted in HA pool to 100, 50, 25, 10, 5, 2.5, 1, 0.5 and 0.1% (v/v) normal in HA canine plasma pool, and underwent analysis using the canine optimized TGT. HA pool was spiked with rcBDDFVIII to 1, 0.5, 0.25, 0.1, 0.05, 0.025, 0.01, 0.005 and 0.001 U/mL final rcBDDFVIII activity. The spiked plasmas were analysed using the canine optimized TGT.

Normal pool II was diluted in the HB plasma pool to 100, 50, 25, 10, 5, 2.5, 1, 0.5 and 0.1% (v/v) normal in HB canine plasma pool, and underwent analysis using the canine optimized TGT.

#### Assessment of imprecision of the canine optimized TGT

Intra assay imprecision estimates were determined by analysis of 20 replicates in the same analytical run of: normal pool II, 0.1% norm. in HA, 0.1% norm. in HB, HA pool and HB pool.

Inter assay imprecision estimates were determined by analysis of duplicates of normal pool I, 0.1% norm. in HA and 0.1% norm. in HB stock over seven separate runs.

#### Assessment of the applicability of the canine optimized TGT to measure thrombin generation in ex vivo samples

Six HA dogs were dosed with rcBDDFVIII or normal canine plasma to achieve FVIII activities of 3, 10 and 100% of normal. Samples were collected 15 and 30 minutes after each dosing as described previously. Between each dosing, a washout period before next administration, lasting at least 14 days, was realized.

The FVIII activity of each sample was measured using a chromogenic FVIII activity assay (COAMATIC FACTOR VIII).

All samples were analyzed using the canine optimized TGT.

### Statistical analysis

All statistical analyses were carried out using GraphPad Prism 6.05 for Windows (GraphPad Software, La Jolla California USA, www.graphpad.com).

Statistical significance assumed for p<0.05.

Differences in peak thrombin generation between normal pooled canine plasma and normal pooled plasma incubated with anti cFVIII PAb analyzed using TGT with 1 and 5 pM TF were analyzed using a Mann-Whitney test (Two-tailed P-value given). The same analysis was carried out when normal pooled canine plasma and HA pooled canine plasma were analyzed under identical conditions.

Difference in peak thrombin generation between a group of five normal dogs and a group of five HA dogs was assessed using the Mann-Whitney test.

When the inhibitory capacity of the anti cFVIII PAb was assessed using the canine optimized TGT, a paired t-test (two-tailed) was used for comparison of peak thrombin generations. The intra-assay CV% for HA plasma was used for the analysis.

Dose dependency between thrombin generation parameters and FVIII or FIX activity was analyzed by plotting raw or transformed thrombin generation parameters against raw or transformed factor activities. The linear associations were calculated as the squared Pearson correlation coefficient r^2^.

When assessing if residual amounts of cFVIII contributed to thrombin generation in HA plasma a multiple paired t-test was used. Intra assay CV% for 0.1% normal in HA was used for this analysis.

Intra-assay imprecision estimates were determined based on 20 replicates of samples. Intra-assay CV%s were divided by 2^½^ to reflect intra-assay imprecision of double determinations.

Acceptability criteria are based upon reported imprecision estimates of human plasma samples analyzed using TGTs, and are presented in S1 Table.

The correlation between log10 transformed FVIII activity plotted against peak thrombin generation was computed as Pearson correlation coefficients (r), given with two-tailed P values. Slopes from plots of log10 transformed FVIII activity against peak thrombin generation (nM) were compared using a one-way ANOVA, and Bartlett’s test was used to test for equal variance among the plots.

Limit of detection for the assay was assessed by comparing CAT parameters obtained from analysis of 20 replicates of HA plasma with 20 replicates of 0.1% (v/v) normal canine plasma in HA or HB plasma using a paired t-test.

## Results

### Inhibitory capacity of an anti-canine-FVIII polyclonal antibody (anti cFVIII PAb)

A residual FVIII activity of 1.3% was observed when the normal canine plasma pool II incubated with 0.1 mg/mL anti cFVIII PAb was analyzed using the chromogenic FVIII activity assay.

### Assessment of thrombin formation in canine plasma samples using the standard CAT method

**Fig 1:**
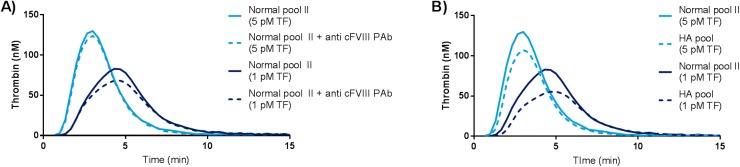
Analysis of normal and haemophilia A canine plasma pools using the CAT method. Thrombin generation was monitored using the standard CAT method, and was initiated using tissue factor at 5 and 1 pM (PPP and PPPlow reagent, respectively). A) Normal plasma pool II was analyzed in the presence and absence of anti cFVIII PAb. B) Normal plasma pool II and HA plasma pool were analyzed. Thrombograms represent mean of quadruplicates. Y-axis depicts thrombin generation in nM, x-axis depicts time in minutes. All experiments were carried out in one run, and the normal plasma pool II results in A) and B) are identical.

A slightly higher thrombin generation (peak thrombin generation) was observed for the normal plasma pool compared with the normal plasma pool incubated with anti cFVIII PAb at both TF concentrations (5 and 1 pM) (Fig 1A) (p>0.05). A higher peak thrombin generation was also observed when normal plasma pool was compared with a HA plasma pool in the CAT setup using 5 and 1 pM TF as trigger (Fig 1B) (p>0.05).

### Analysis of canine plasma material using the canine optimized TGT

#### Assessment of the capability of the canine optimized TGT to discriminate between normal and HA canine plasma

**Fig 2:**
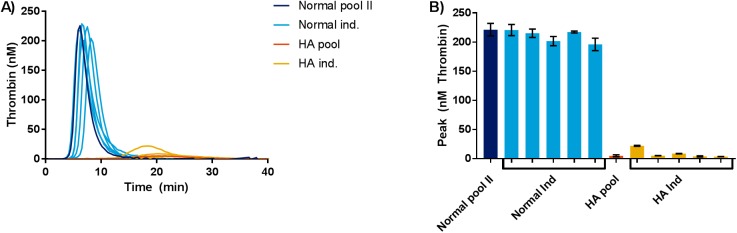
Analysis of normal and HA plasma using the canine optimized TGT. Plasma samples from five healthy dogs, five HA dogs, a normal canine plasma pool and a HA canine plasma pool were analyzed. A) Thrombograms represent mean of double determinations. Y-axis depicts thrombin generation in nM, x-axis depicts time in minutes. B) Bars represent mean peak thrombin generation in nM, and error bars represent range of double determinations. Y-axis depicts peak thrombin generation in nM, and x-axis depicts samples.

Peak thrombin activities spanning 200–230 nM thrombin were observed for the normal dogs and the normal pool, whereas HA plasma samples gave rise to peak thrombin activities spanning 5–25 nM thrombin. Both lag time and TTP were prolonged for the HA samples compared with the normal, and the ETP of the HA samples was decreased as well (Fig 2). A statistically significant difference was observed in the peak thrombin generations between normal and HA canine plasma (p<0.05).

**Fig 3:**
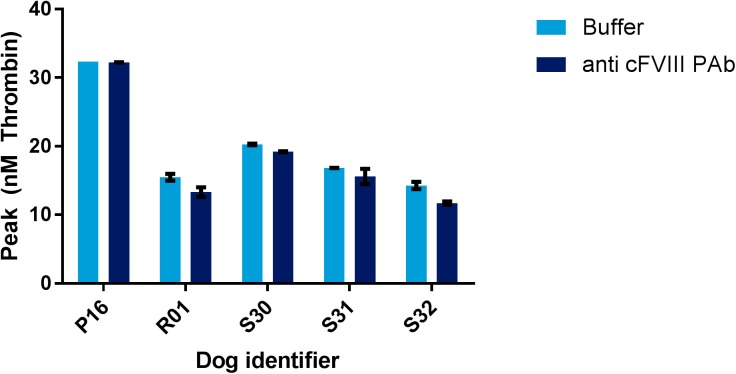
Analysis of plasma samples from five individual HA dogs using the canine optimized TGT. Bars represent mean of double determinations, and error bars represent range. Light blue bars represent analysis of plasma samples in the presence of buffer, and dark blue bars represent analysis of plasma samples in the presence of 0.1 mg/mL anti cFVIII PAb. The buffer sample from P16 represents a single determination, as one of the double determinations failed. X-axis represents dog identifier, and y-axis represents peak thrombin generation in nM thrombin.

It was tested if residual amounts of FVIII gave rise to inter-individual variation in thrombin generation in HA dogs. As mentioned previously, all plasma samples collected are from severe HA dogs, and present with FVIII activity levels below 0.3%, as judged by chromogenic FVIII activity analysis (COAMATIC FACTOR VIII). No significant difference in peak thrombin generation was observed between individual canine HA samples in the presence or absence of 0.1 mg/mL anti cFVIII PAb (p>0.05) (Fig 3).

When confirming the capacity of the anti cFVIII PAb to inhibit thrombin formation in plasma a statistically significant decreased thrombin generation was observed when normal pool was pre-incubated with 0.1 mg/mL anti cFVIII PAb. No difference was observed between peak thrombin generation of normal pool incubated with anti cFVIII PAb and peak thrombin generation of the HA pool (p>0.05) (S1 Fig).

#### Assessment of the cFVIII and cFIX dose dependent thrombin generation

**Fig 4:**
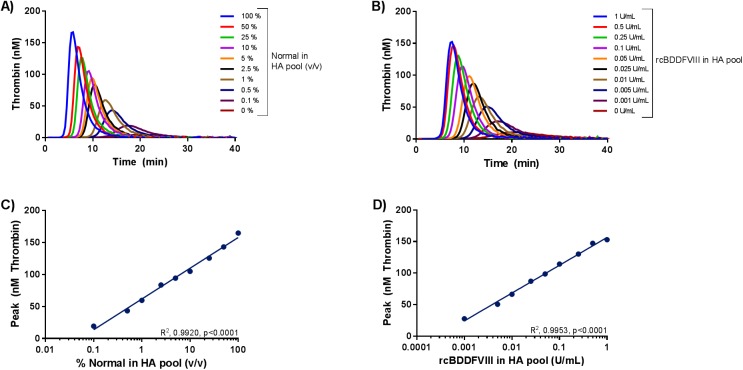
Analysis of normal canine plasma pool and rcBDDFVIII dilutions in haemophilia A canine plasma pool. Samples were analyzed using the canine optimized thrombin generation test. A) Dilutions spanning 0–100% normal in HA plasma pool (v/v) were analyzed. 100% represents undiluted normal pool and 0% represents undiluted HA plasma pool. Normal pool I was utilized. B) The HA plasma pool was spiked with 0–1 U/mL rcBDDFVIII. Zero U/mL represents HA plasma. Thrombograms represent mean of double determinations. Y-axis depicts thrombin generation in nM, x-axis depicts time in minutes. C) Correlation analysis of peak thrombin generation versus dilution of normal canine plasma pool in HA pool from panel A. D) Correlation analysis of peak thrombin generation versus rcBDDFVIII activity spiked into the HA canine plasma pool from panel B. The log10 transformed x-axes in panel C and D represent plasma dilutions or rcBDDFVIII activity, and y-axes represent peak thrombin generation in nM. Points represent mean of double determinations, lines represent linear regression analysis.

cFVIII dose dependent thrombin generation was observed when a dilution series of normal pool I spanning 0–100% (v/v) normal in HA pool or rcBDDFVIII (0-1U/mL) spiked in HA pool were analyzed using the canine optimized TGT (Fig 4). Dose dependency was observed for all four CAT parameters, (r^2^ >0.98, p<0.05 for all correlations). (Fig 4, S2 Fig and S3 Fig)

**Fig 5:**
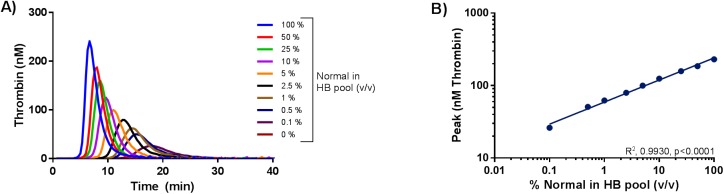
Analysis of normal canine plasma pool dilutions in haemophilia B canine plasma pool. Samples were analyzed using the canine optimized thrombin generation test. A) Dilutions spanning 0–100% normal diluted in HB plasma pool (v/v) were analyzed. 100% represents undiluted normal plasma pool, and 0% represents undiluted HB pool. Thrombograms represent mean of double determinations. Y-axis depicts thrombin generation in nM, x-axis depicts time in minutes. B) Correlation analysis of peak thrombin generation versus dilution of normal canine plasma pool in HB pool from panel A. The log10 transformed x-axis represents plasma dilutions, and the log10 transformed y-axis represents peak thrombin generation in nM. Points represent mean of double determinations, lines represent linear regression analysis.

Assay sensitivity towards cFIX was assessed by diluting normal pool in a HB plasma pool (Fig 5). A statistical significant dose dependent relationship between dilution degree and thrombin generation was observed for all four CAT parameters (r^2^ >0.96, p<0.05 for all correlations) (Fig 5 and S4 Fig).

#### Assessment of imprecision of the canine optimized TGT

**Fig 6:**
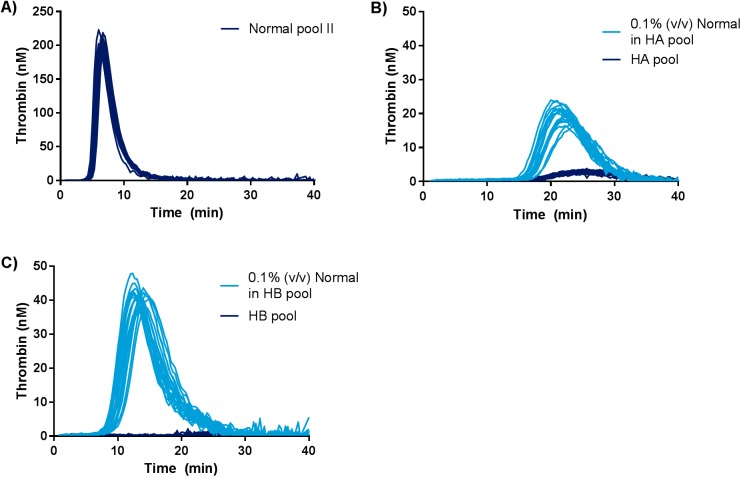
Thrombograms from imprecision estimation of the canine optimized TGT. Twenty replicates of each sample were analyzed per run. Each thrombogram represents a single determination. The following samples were analyzed: A) normal canine plasma pool II, B) normal canine plasma pool II diluted to 0.1% (v/v) in haemophilia A (HA) plasma pool and the HA plasma pool, C) normal canine plasma pool II diluted to 0.1% (v/v) in haemophilia B (HB) plasma pool and the HB plasma pool. Y-axis depicts thrombin generation in nM and x-axis depicts time in minutes.

Thrombograms from intra-assay imprecision runs are presented in Fig 6, and imprecision estimates are presented in Table 1. Inter-assay imprecision estimates are given in Table 2. The intra- and inter-assay imprecision of the assay is acceptable according to the criteria established in the statistical section and S1 Table.

**Table 1:** Intra-assay imprecision estimates.

	**Normal pool II**		**0.1% norm in HA**		**0.1% norm in HB**		**HA pool**	
	Mean	CV%	Mean	CV%	Mean	CV%	Mean	CV%
**Lag time**	5	3.2	16.9	2.6	9.2	5.2	16.8	3.7
**ETP**	700.6	1.9	175.6	5.7	308.9	3.8	46.4	16.5
**Peak**	201.2	2.5	19.8	8.1	41.4	4.0	2.2	14.9
**Time to peak**	6.7	2.5	21.9	2.8	13.2	4.3	26.1	4.4

The table presents intra-assay imprecision estimates of double determinations based upon analysis of 20 replicates of a normal canine plasma pool, 0.1% (v/v) normal canine plasma pool in a haemophilia A (HA) canine plasma pool, 0.1% (v/v) normal canine plasma pool in a haemophilia B (HB) canine plasma pool and a HA canine plasma pool. Values are given in Lag time; min, ETP; nM·min, Peak; nM, time to peak; min. CV%; coefficient of variation given as percentage.

**Table 2:** Inter-assay imprecision estimates.

	**Normal pool I**		**0.1% norm in HA**		**0.1% norm in HB**	
	Mean	CV%	Mean	CV%	Mean	CV%
**Lag time**	4.8	8.6	14.6	9.2	13.1	7.6
**ETP**	683.7	4.5	268.5	9.4	240.9	13
**Peak**	167.4	7.7	32	13.2	33.3	15.3
**Time to peak**	6.9	8	19.2	6.8	17.4	7.1

The table presents inter-assay imprecision estimates based upon analysis of duplicates of a normal canine plasma pool, 0.1% (v/v) normal canine plasma pool in a haemophilia A (HA) canine plasma pool and 0.1% (v/v) normal canine plasma pool in a haemophilia B (HB) canine plasma pool in seven independent runs. Values are given in Lag time; min, ETP; nM*min, Peak; nM, time to peak; min. CV%; coefficient of variation given as percentage.

#### Limit of detection

From imprecision data it was demonstrated that the ETP, Peak thrombin generation and TTP parameters were significantly different between deficient plasma and 0.1% (v/v) normal in deficient plasmas (p<0.01). Limit of detection of the assay is therefore set to ≤0.1% (v/v) normal in HA or HB plasma.

#### Assessment of the applicability of the canine optimized TGT to measure thrombin generation in ex vivo samples

**Fig 7:**
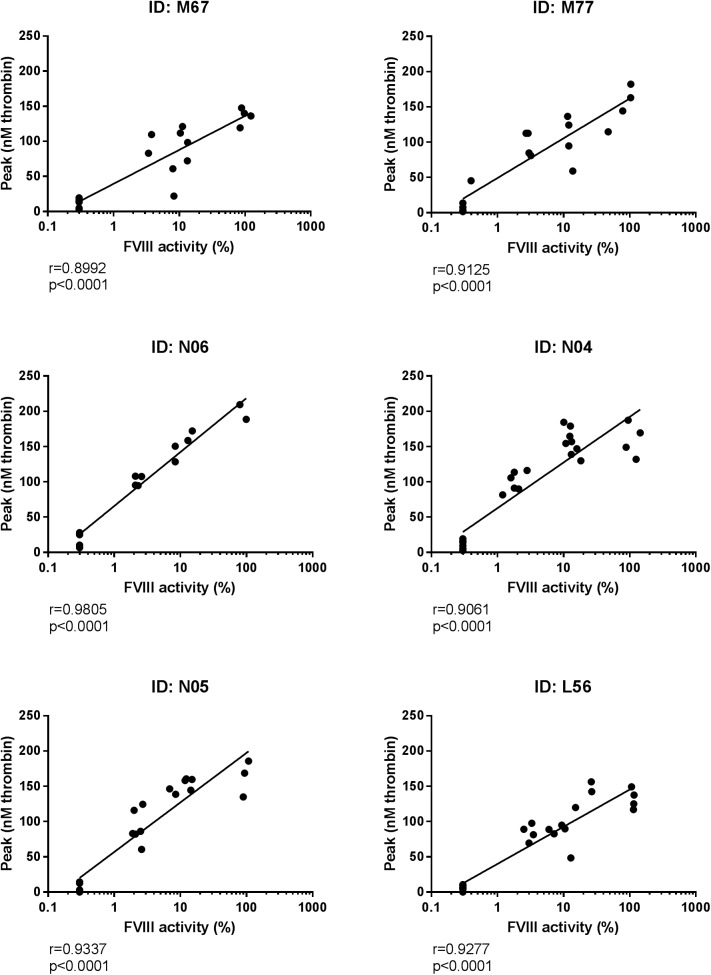
Correlation between FVIII activity and thrombin generation in *ex vivo* plasma samples. Correlation analysis between FVIII activities and peak thrombin generation of samples from six individual haemophilia A dogs dosed with varying levels of rcBDDFVIII and normal canine plasma on several occasions. Each point represents mean of double determinations, and the line represents linear regression analysis of the plots. Y-axes depict peak thrombin generation in nM thrombin, and the log10 transformed x-axes depict FVIII activity given as percentage of FVIII activity in a normal canine plasma pool. IDs represent the identifiers for each dog, r represents Pearson correlation coefficient, and P represents two-tailed P values.

Correlations between FVIII activity measurement and peak thrombin generation from six individual dogs are depicted in Fig 7. Statistically significant correlations were observed between log10 transformed FVIII activities and peak thrombin generation for all six dogs. Pearson correlation coefficients (r) spanned 0.90–0.98, all with P-values<0.0001. Slopes from the plots of log10 transformed FVIII activity against peak thrombin generation were compared, and were statistically significantly different (p<0.0001, one-way ANOVA). Equal variance of the calculated slopes were confirmed (p = 0.47, Bartlett’s test).

All raw data is available in S1 File.

## Discussion

With the canine optimized TGT a clear differentiation between normal and HA canine plasma was possible (Fig 2). Inter-individual differences in thrombin generation potential were observed both for individual normal dogs and individual HA dogs, which has also been observed among healthy individuals and HA patients [18, 19]. It was ruled out that the inter-individual variability in the thrombin generation potential observed in five individual HA dogs was related to residual amounts of FVIII in the plasma samples, by analysing samples in the presence and absence of an anti cFVIII PAb (Fig 3). The present assay is dose dependently sensitive to both the FVIII and FIX levels in canine plasma, as dilutions of normal in deficient plasma and spike with rcBDDFVIII in HA plasma gave rise to dose dependent thrombin generation (Figs 4 and 5 and S2–S4 Figs).

The limit of detection of the assay was at least 0.1% FVIII and 0.1% FIX (Fig 6) and intra- and inter assay imprecision analysis revealed acceptable CV percentages both for normal canine plasma and for 0.1% (v/v) normal in deficient plasmas (Table 1 and Table 2).

The applicability of the assay for analysis of thrombin generation *ex vivo* was assessed by analysis of plasma samples collected from six severe HA dogs treated with rcBDDFVIII or normal canine plasma. Correlations between measured plasma FVIII activity and peak thrombin generation were observed for all six dogs (Fig 7). This agreement justifies utilization of the assay both for *in vitro* and *ex vivo* assessment of FVIII compound efficacy in dogs.

Within the field of haemophilia research TGT is widely used for *in vitro* and *ex vivo* assessment of drug efficacy in animal models of haemophilia[12, 20]. Typically thrombin generation is triggered by activation of the extrinsic pathway of coagulation with rhTF. However, when we analyzed plasma samples from normal and severe HA dogs using rhTF activation no usable difference in thrombin generation was observed (Fig 1). Therefore we developed a canine optimized TGT, where thrombin generation was triggered via the intrinsic pathway of coagulation using the APTT reagent Actin FS. Actin FS was selected as trigger based upon its synthetic and well defined composition, supposedly assuring less lot-to-lot variation compared with other APTT reagents where purified natural sources e.g. rabbit brain extract are part of the reagent.

During the *ex vivo* studies we observed a difference in response to FVIII or plasma treatment measured as peak thrombin generation among the six HA dogs (Fig 7). It was evident that the correlation between measured FVIII activity and peak thrombin generation differed between the dogs and this even though all six dogs carried the same F8 mutation. These observations raise the question whether the assay would be suitable for individualization of FVIII treatment, and if the variability in thrombin generation response to treatment correlates with clinical response to treatment. Further studies are needed to answer these questions.

When measuring baseline thrombin generation potential in five individual dogs, inter-individual differences were observed in their thrombin generation capacity (Fig 3). All dogs presented with undetectable FVIII activity leves (<0.3% in Chromogenic assay), and carry the same F8 mutation. Whether or not the inter-individual variability in thrombin generation capacity relates to the clinical bleeding phenotype remains unknown, and warrants further studies.

Compared with other global coagulation methods, e.g. thromboelastography, where freshly drawn whole blood is required, the present method excels as it allows high capacity measurement of frozen plasma samples (30+ samples/run). This renders the method suitable for larger pre-clinical studies, which otherwise would be more laborious and time consuming.

## Conclusion

In conclusion we established a TGT highly sensitive to the levels of FVIII and FIX in canine plasma, which allowed us to distinguish between normal and haemophilia dogs. Clear dose dependent correlations were observed between factor activities and thrombin generation parameters, and a solid agreement was observed between *in vitro* and *ex vivo* efficacy of FVIII. Imprecision estimation of the assay revealed a robust assay that allows reliable monitoring of thrombin generation in plasma from haemophilia dogs.

## Supporting information

S1 Fig Analysis of a normal plasma pool +/- anti canine FVIII PAb and HA pool. Normal canine plasma pool was analyzed using the canine optimized TGT in the presence and absence of anti cFVIII PAb, HA plasma pool was included as a control. All samples were analyzed in duplicate, and thrombograms represent mean of double determinations. Y-axis depicts thrombin generation in nM, x-axis depicts time in minutes.
(TIF)

S2 Fig Correlation analysis between CAT parameters and normal plasma in HA plasma dilutions. Correlation analysis of calibrated automated thrombogram (CAT) parameters obtained from analysis of normal canine plasma pool diluted in haemophilia A plasma pool, versus dilution of normal canine plasma pool in HA canine plasma pool. Points represent mean of double determinations, lines represent linear regression analysis. The log10 transformed x-axes represent plasma dilutions, and y-axes represent CAT parameters. ETP; Endogenous Thrombin Potential.
(TIF)

S3 Fig Correlation analysis between CAT parameters and rcBDDFVIII activity in HA plasma. Correlation analysis of calibrated automated thrombogram (CAT) parameters obtained from analysis of HA canine plasma pool spiked with rcBDDDFVIII, versus rcBDDFVIII activity spiked into the HA canine plasma pool. Points represent mean of double determinations, lines represent linear regression analysis. The log10 transformed x-axes represent U/mL rcBDDFVIII in HA plasma, and y-axes represent CAT parameters. ETP; Endogenous Thrombin Potential.
(TIF)

S4 Fig Correlation analysis between CAT parameters and normal plasma in haemophilia B plasma dilutions. Correlation analysis of calibrated automated thrombogram (CAT) parameters obtained from analysis of normal canine plasma pool diluted in haemophilia B (HB) plasma pool, versus dilution of normal canine plasma pool in HB canine plasma pool. Points represent mean of double determinations, lines represent linear regression analysis. The log10 transformed x-axes represent plasma dilutions, and y-axes represent CAT parameters (log10 transformed y-axes for ETP). ETP; Endogenous Thrombin Potential.
(TIF)

S1 File Raw data for all figures. In this compressed file all raw data is available in excel format. Data is sorted according to figure.
(ZIP)

S1 Table Acceptability criteria for imprecision estimates. Lag; Lag time, ETP; endogenous thrombin potential, Peak; peak thrombin generation, TTP; time to peak. ^*^ and ^#^ indicate that the criteria were established from Spronk et al [1]. or Waters et al. [2] respectively.
(DOCX)
