# Supplementary figures and images for: Global measurement of coagulation in plasma from normal and haemophilia dogs using a novel modified thrombin generation test – Demonstrated in vitro and ex vivo
Source: PLoS One. 2017 Apr 6;12(4):e0175030. doi: 10.1371/journal.pone.0175030 (PMC5383133; doi:10.1371/journal.pone.0175030)

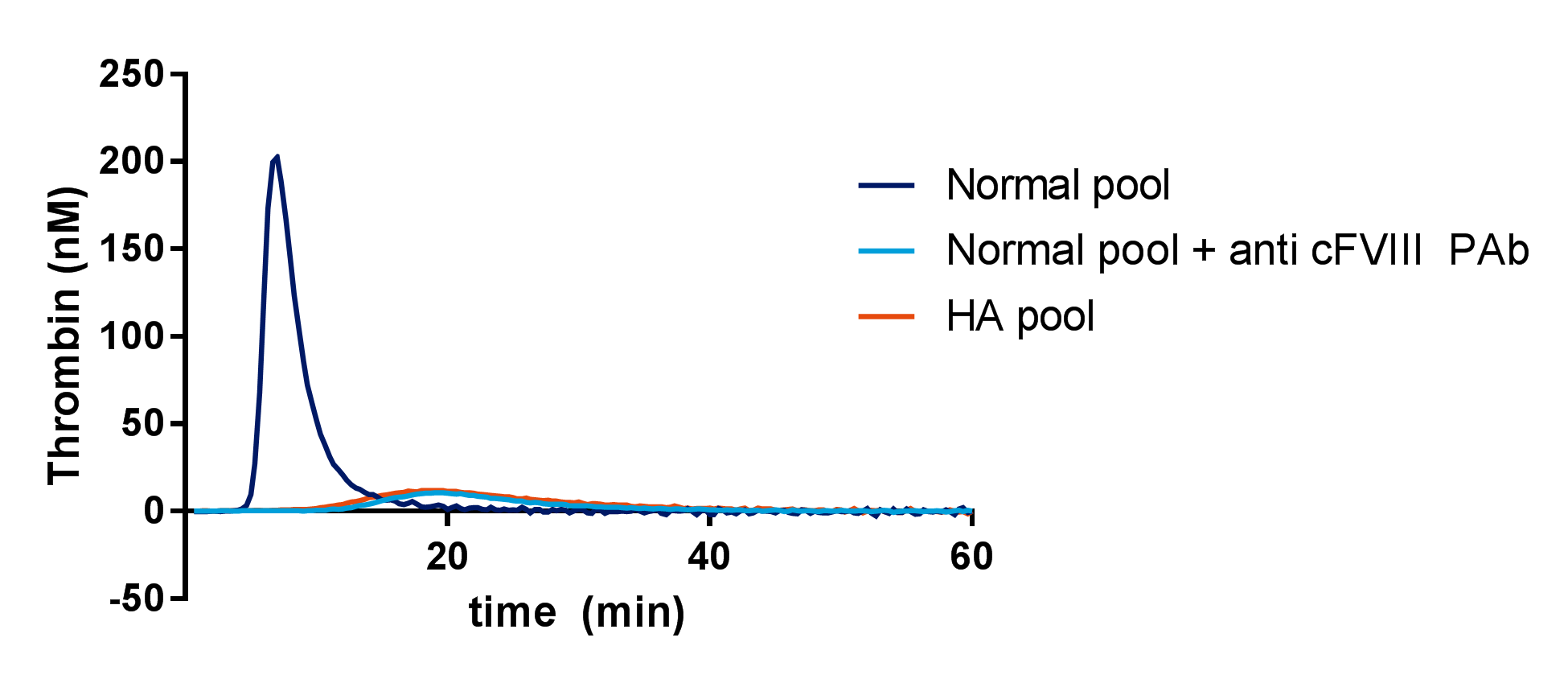

Supplement: S1 Fig — Normal canine plasma pool was analyzed using the canine optimized TGT in the presence and absence of anti cFVIII PAb, HA plasma pool was included as a control. All samples were analyzed in duplicate, and thrombograms represent mean of double determinations. Y-axis depicts thrombin generation in nM, x-axis depicts time in minutes. (TIF) [file pone.0175030.s001.tif]

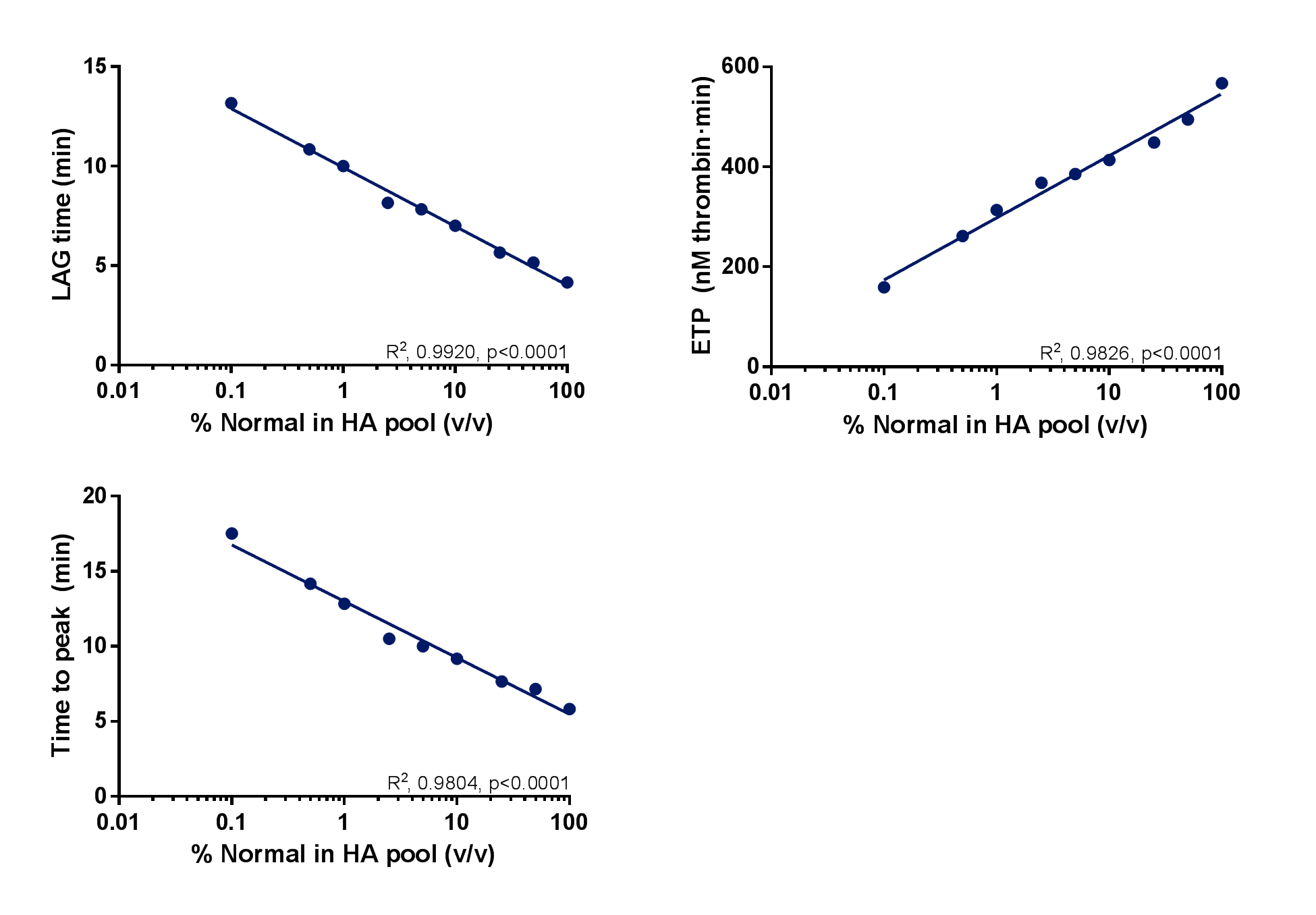

Supplement: S2 Fig — Correlation analysis of calibrated automated thrombogram (CAT) parameters obtained from analysis of normal canine plasma pool diluted in haemophilia A plasma pool, versus dilution of normal canine plasma pool in HA canine plasma pool. Points represent mean of double determinations, lines represent linear regression analysis. The log10 transformed x-axes represent plasma dilutions, and y-axes represent CAT parameters. ETP; Endogenous Thrombin Potential. (TIF) [file pone.0175030.s002.tif]

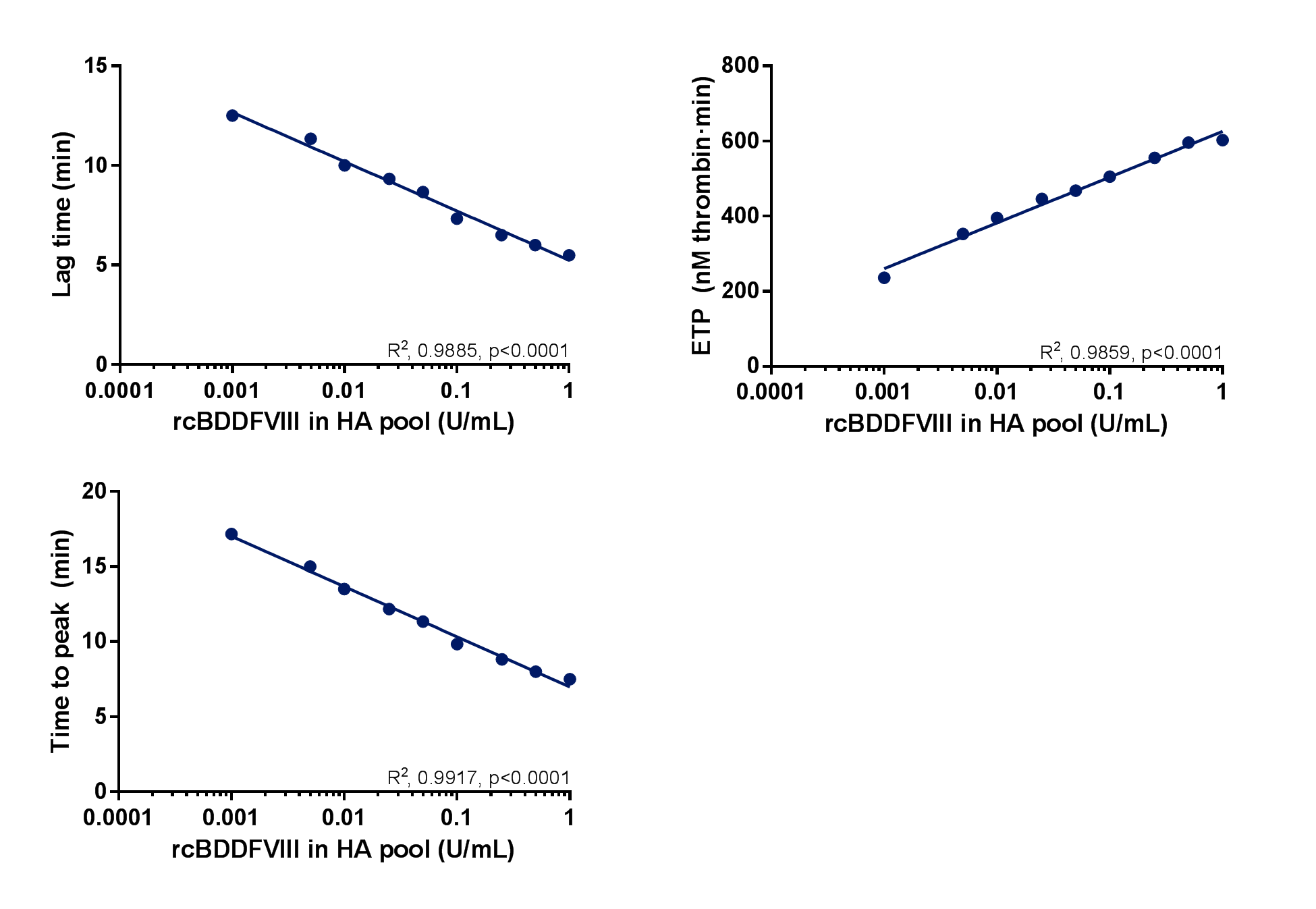

Supplement: S3 Fig — Correlation analysis of calibrated automated thrombogram (CAT) parameters obtained from analysis of HA canine plasma pool spiked with rcBDDDFVIII, versus rcBDDFVIII activity spiked into the HA canine plasma pool. Points represent mean of double determinations, lines represent linear regression analysis. The log10 transformed x-axes represent U/mL rcBDDFVIII in HA plasma, and y-axes represent CAT parameters. ETP; Endogenous Thrombin Potential. (TIF) [file pone.0175030.s003.tif]

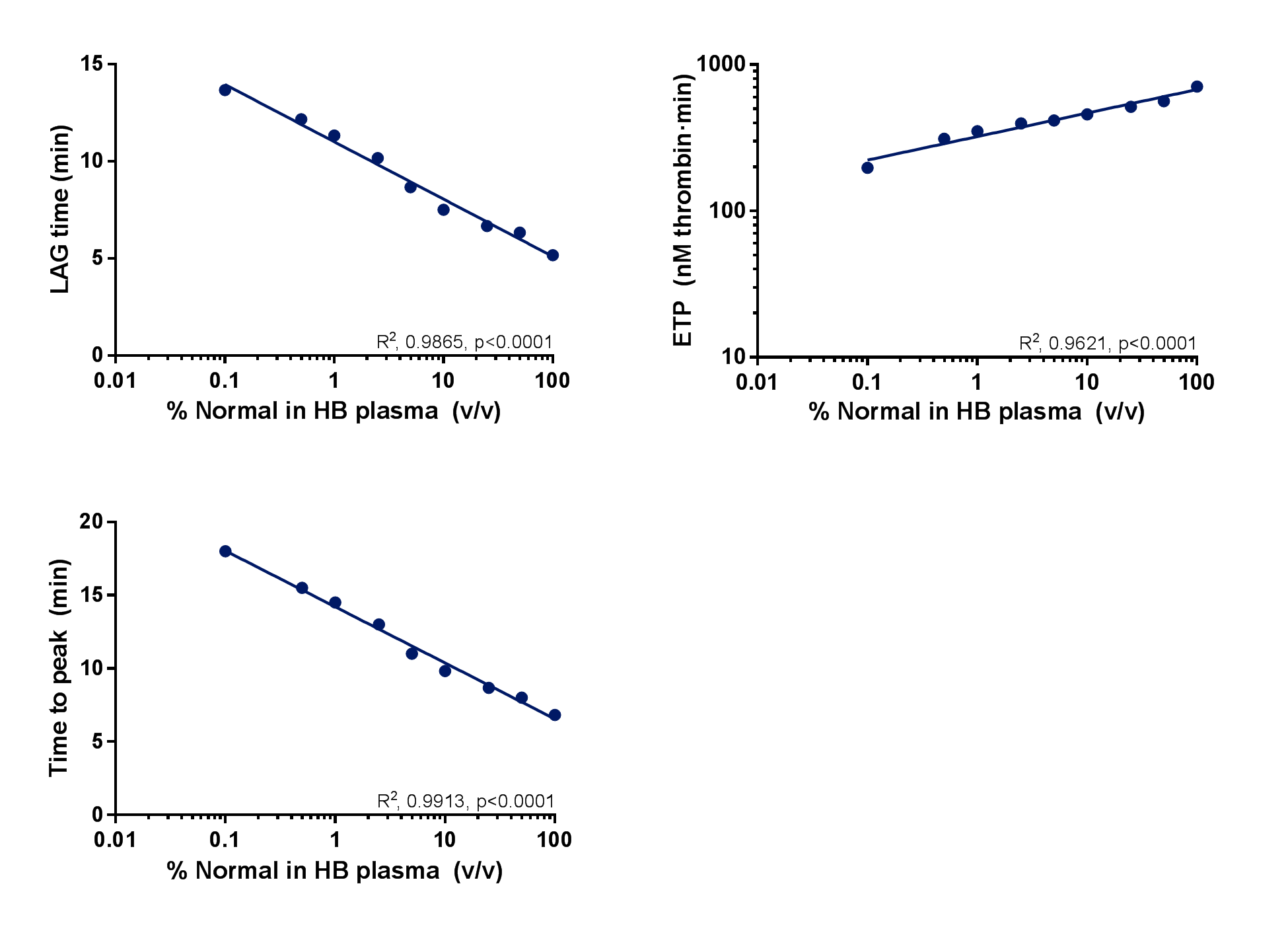

Supplement: S4 Fig — Correlation analysis of calibrated automated thrombogram (CAT) parameters obtained from analysis of normal canine plasma pool diluted in haemophilia B (HB) plasma pool, versus dilution of normal canine plasma pool in HB canine plasma pool. Points represent mean of double determinations, lines represent linear regression analysis. The log10 transformed x-axes represent plasma dilutions, and y-axes represent CAT parameters (log10 transformed y-axes for ETP). ETP; Endogenous Thrombin Potential. (TIF) [file pone.0175030.s004.tif]
