# Supplementary material for: Global measurement of coagulation in plasma from normal and haemophilia dogs using a novel modified thrombin generation test – Demonstrated in vitro and ex vivo
Source: PLoS One. 2017 Apr 6;12(4):e0175030. doi: 10.1371/journal.pone.0175030 (PMC5383133; doi:10.1371/journal.pone.0175030)
Supplement: S1 Table — Lag; Lag time, ETP; endogenous thrombin potential, Peak; peak thrombin generation, TTP; time to peak. * and # indicate that the criteria were established from Spronk et al [1]. or Waters et al. [2] respectively. (DOCX) [file pone.0175030.s006.docx]

|  | Intra-assay | | | | |  | Inter-assay | | |
| --- | --- | --- | --- | --- | --- | --- | --- | --- | --- |
|  | | **Lag** | **ETP** | **Peak** | **TTP** | **Lag** | **ETP** | **Peak** | **TTP** |
| Normal plasma^*^ | | ≤6% | ≤6% | ≤6% | ≤6% | ≤10% | ≤10% | ≤10% | ≤10% |
| 0.1% Norm in HA^#^ | | N/A | ≤13.1% | ≤17.1% | N/A | N/A | N/A | N/A | N/A |
| 0.1% Norm in HB^#^ | | N/A | ≤31.6% | ≤34.2% | N/A | N/A | N/A | N/A | N/A |
| HA^#^ | | N/A | ≤26.2% | ≤23.6% | N/A | N/A | N/A | N/A | N/A |

**References**

1. Spronk HMH, Dielis AWJH, De Smedt E, van Oerle R, Fens D, Prins MH, et al. Assessment of thrombin generation II: Validation of the Calibrated Automated Thrombogram in platelet-poor plasma in a clinical laboratory. Thrombosis and haemostasis. 2008.

2. Waters EK, Hilden I, Sorensen BB, Ezban M, Holm PK. Thrombin generation assay using factor XIa to measure factors VIII and IX and their glycoPEGylated derivatives is robust and sensitive. Journal of thrombosis and haemostasis : JTH. 2015;13(11):2041-52.
